# DNA-Binding Motif of the Imprinted Transcription Factor PEG3

**DOI:** 10.1371/journal.pone.0145531

**Published:** 2015-12-21

**Authors:** Suman Lee, An Ye, Joomyeong Kim

**Affiliations:** Department of Biological Sciences, Louisiana State University, Baton Rouge, Louisiana, 70803, United States of America; University of Connecticut, UNITED STATES

## Abstract

*Peg3* is an imprinted gene that is predicted to encode a DNA-binding zinc finger protein. This was previously demonstrated through Chromatin ImmunoPrecipitation-based Sequencing experiments. In the current study, we reanalyzed the previous ChIP-Seq results and further characterized the DNA-binding motif of PEG3. According to the results, PEG3 binds to the promoters and enhancers of a subset of genes that are closely associated with the known functions of *Peg3*. Some of these identified targets include *Tufm*, *Mrpl45*, *Cry2*, *Per1*, *Slc25a29* and *Slc38a2*. With this set of targets, we derived a DNA-binding motif of PEG3, 5’-GTGGCAGT-3’, which also provides a tabulated matrix that can be used for predicting other unknown genomic targets. Among the newly identified targets, we analyzed in detail the two loci, *Slc38a2* and *Slc38a4*, which are known to be involved in neutral amino acid transport. The results indicated that PEG3 likely functions as a transcriptional repressor for these two loci. Overall, the current study provides a set of genomic targets and also redefines the DNA-binding motif for the imprinted transcription factor PEG3.

## Introduction

*Peg3* (paternally expressed gene 3) is an imprinted gene localized in human chromosome 19q13.4/proximal mouse chromosome 7 [1–3]. *Peg3* is expressed mainly from the paternal allele, and the maternal allele is repressed by DNA methylation [1]. *Peg3* is highly expressed in placenta and brains of the animals [1,3], and mutagenesis experiments have confirmed critical roles played by *Peg3* in controlling fetal growth rates and maternal caring behaviors [4,5]. *Peg3* is well conserved among placental mammals, but the orthologous sequences of this gene have not been identified beyond the eutherian mammals, suggesting that *Peg3* may be a newly derived gene in this lineage [6,7]. Human *PEG3* is quite often identified as a potential tumor suppressor based on the observation that the promoter region of *PEG3* is usually hypermethylated in the patients of ovarian and breast cancers [8,9]. However, the actual mechanism by which *PEG3* contributes to cancers is currently unknown.

*Peg3* is localized in the middle of a gene cluster that is known to encode C2H2, Kruppel-type zinc finger proteins [3]. In fact, *Peg3* itself is predicted to encode this type of proteins based on the detection of 12 zinc finger motifs within its ORF (Open Reading Frame) [2,3]. A series of recent studies further confirmed this predicted function of PEG3 [10,11]. According to these studies, PEG3 binds to a large number of genomic targets as a DNA-binding protein, and its consensus DNA-binding motif is as follows: 5’-AGTnnCnnnTGGCT-3 with ‘n’ indicating any nucleotide base [10]. Detailed experiments further demonstrated that PEG3 likely functions as a transcriptional repressor for the downstream target genes [10,11]. Consistent with this, genome-wide expression analyses indicated that several placenta-specific gene families are up-regulated in the brains of the mutant mouse lacking PEG3 [5]. Interestingly, the majority of these up-regulated genes are usually marked with the histone modification H3K9me3 (trimethylation on lysine 9 of histone 3) [12,13]. Thus, it has been hypothesized that PEG3 may repress its downstream genes through H3K9me3-mediated mechanisms [5,11].

As part of ongoing efforts to characterize the protein function of PEG3, we have analyzed our previous ChIP-Seq results more carefully in the current study. The main motivation for this analysis stems from the realization that this particular set of ChIP-Seq results has not been properly analyzed with appropriate controls. Thus, we analyzed again this set of ChIP-Seq results, providing a set of new genomic targets. With this new set of genomic targets, we further defined a DNA-binding motif for PEG3: 5’-GTGGCAGT-3’. We also tested the binding and subsequent functional connection of PEG3 to a couple of new targets, *Slc38a2* and *Slc38a4*. The detailed results are described below.

## Results

### Reanalysis of the ChIP-Seq results of PEG3

In the current study, we reanalyzed our ChIP-Seq results that had been derived from brains in the following manner. This set of ChIP-Seq results was previously processed without proper controls, thus generating a set of potential binding sites of PEG3 with uncertain statistical significance [10]. Thus, we performed another two sets of NGS (Next Generation Sequencing) runs using the input DNA and the immunoprecipitated DNA with pre-immune serum, and subsequently used these sets of results as controls for predicting the binding sites for PEG3. After the proper processing of ChIP-Seq results, which has been described in detail in Materials and Methods, we were able to obtain 80 potential binding sites (**S1 Table**). According to an initial inspection, 56 binding sites are closely associated with genes whereas the remaining 24 sites are localized in the intergenic regions. Out of 56 gene-associated binding sites, ChIP-Seq peaks at the 21 sites were statistically significant, and also the majority of these 21 binding sites are localized within either the promoters or enhancer regions of the 19 individual genes (**Table 1**). Among these genes, the following four genes have been identified again as the potential target genes of PEG3 as reported previously, including *Pgm2l1* (phosphoglucomutase 2 like 1), *Tufm* (elongation factor Tu, mitochondrial form 1), *Dusp1* (dual specificity protein phosphatase 1) and *Mrpl45* (39S ribosomal protein L45, mitochondrial). This indicates that some of the previously identified binding sites may be genuine targets of PEG3. Among the newly identified genes, the following are noteworthy given the known functions of PEG3. First, *Peg3* has been recently identified as a major regulator for autophagy [14], and one of the PEG3 binding sites is derived from a 3’-side enhancer of *Pik3c3* (phosphatidylinositol 3-kinase catalytic subunit), a well-known gene involved in autophagy [15,16]. Second, the two binding sites of PEG3 are associated with the promoters of the two genes that are important for circadian rhythm, *Cry2* (cryptochrome 2) and *Per1* (period circadian protein homolog 1). This is interesting since some of imprinted genes are known to be involved in controlling circadian rhythm [17]. Third, three binding sites are associated with one particular gene, *Slc38a2* (sodium-coupled neutral amino acid transporter 2): two binding sites are from a 5’ enhancer while one site from the promoter. The association of the three binding sites with one particular gene is very unusual given the overall small number of the predicted binding sites of PEG3, totaling 80 binding sites. Thus, the potential binding of PEG3 to this gene has been analyzed further in the later section of the current study. Overall, this series of analyses identified 80 potential genomic targets of PEG3, and 21 of these sites are associated with 19 individual genes with potential functional connections to PEG3.

**Table 1:** Potential genomic targets of PEG3 in the mouse genome (mm9).

chr	start	end	gene	position
chr2	92274630	92275019	Cry2	promoter
chr2	165835510	165836159	Ncoa3	enhancer (1st intron)
chr4	134039455	134040223	Paqr7	5' enhancer
chr7	133630530	133631055	Tufm	promoter
chr7	107375450	107376157	Pgm2l1	promoter
chr10	79817950	79818511	Plk5	Promoter
chr10	84488497	84489095	Ric8b	3' enhancer
chr11	68908354	68908976	Per1	promoter
chr11	97176776	97177720	Mrpl45	promoter
chr12	110071063	110071473	Slc25a29	promoter
chr14	20902311	20902958	Saysd1	promoter
chr15	9070038	9070407	Skp2	promoter
chr15	96528673	96530275	Slc38a2	promoter
chr15	96539404	96539914	Slc38a2	5' enhancer
chr15	96540631	96541033	Slc38a2	5' enhancer
chr16	92158957	92159769	Ak087806	enhancer (1st intron)
chr17	26641241	26641976	Dusp1	promoter
chr18	30689583	30690271	Pik3c3	3' enhancer
chr19	5811752	5812198	Malat1	3' enhancer
chr19	5844399	5846500	Neat1	promoter
chr19	53444058	53444725	Mxi1	enhancer (intron)

### Characterization of the DNA binding motif of PEG3

**Fig 1:**
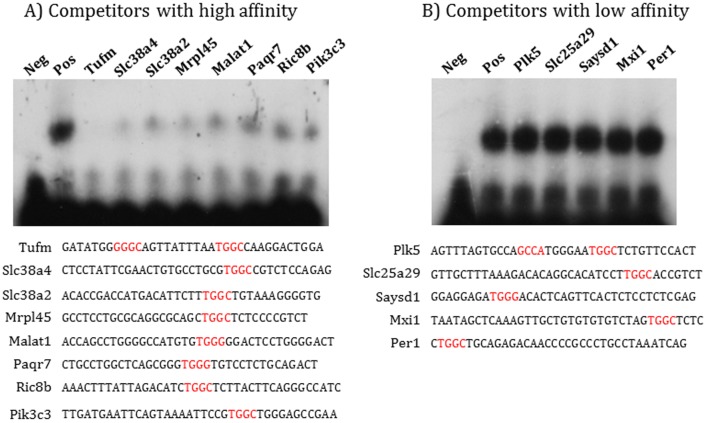
DNA-binding affinity of two sets of competitors. The genomic regions identified through ChIP-Seq were examined to identify potential DNA-binding sites for PEG3 with its known core motif, 5’-TGGC-3’, which is marked in red. These selected regions were competed against the ^32^P-labeled Pgm2l1 oligonucleotide duplex. These assays derived two types of sequences: the competitors with high (**A**) and low (**B**) affinity. The labels for the pictures are as follow: Neg, a negative control without nuclear extract; Pos, a positive control with nuclear extract. The gene names in the remaining lanes indicate the oligonucleotide duplexes that have been tested as competitors at 100x molar ratio to the Pgm2l1 probe.

PEG3 is known to bind to the following consensus DNA-binding motif: 5’-AGTnnCnnnTGGCT-3’ with ‘n’ indicating any nucleotide bases [10]. In the current study, we have further characterized this DNA-binding motif of PEG3 with a more systematic approach as described below. According to the previous study, one small sequence motif, 5’-TGGC-3’ or 5’-TGTC-3’ in some cases, within the 14-bp-long degenerate consensus sequence has been shown to be very critical for PEG3 binding [10]. We manually inspected the genomic sequences of the 21 target regions that are presumably bound by PEG3, and subsequently identified 24 small regions surrounding the 5’-TGGC-3’ motif (**S1 File**). The sequences of these individual regions were tested against the sequence of a known target region of PEG3, *Pgm2l1*, using a series of gel shift assays (**Fig 1**). As shown in lane 2 of **Fig 1**, the P^32^-labeled oligonucleotide duplex probe of *Pgm2l1* was bound and shifted by a complex containing the protein PEG3, which was previously confirmed through supershift assays [10]. A set of 24 oligonucleotide duplexes were subsequently competed against the Pgm2l1 probe. The representative results from 13 duplexes are shown in **Fig 1** and those from the remaining duplexes are also summarized in **S1 File**. According to the results, 12 oligonucleotide duplexes strongly competed with the Pgm2l1 probe, indicating that these regions likely contain DNA-binding sites for PEG3 (**Fig 1A**). Interestingly, the remaining 12 oligonucleotide duplexes didn’t compete at all against the Pgm2l1 probe though they all had the critical core motif, 5’-TGGC-3’ (**Fig 1B**). This suggests that some other unknown motifs may also be critical for PEG3 binding besides the known motif, 5’-TGGC-3’.

**Fig 2:**
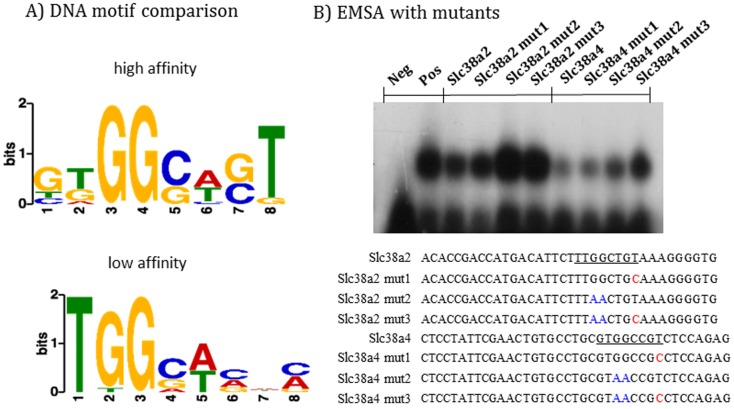
Comparison of the competitors with high and low affinity. (**A**) The competitors with high and low affinity were individually analyzed using the MEME program. This motif search derived two similar but slightly different motifs: GTGGCAGT and TGGCACnC. (**B**) Two individual competitors with high affinity were further analyzed with their mutated versions of competitors. The GG-to-AA mutation is marked in blue whereas the T-to-C mutation is marked with red. The competition was performed with each duplex against the Pgm2l1 probe at the molar ratio of 100 to 1.

To further confirm the above possibility, we performed a series of motif analyses using the two sets of DNA sequences: the competitors with high and low affinity (**Fig 1**). According to the results derived from MEME analyses [18,19], each set of competitors contains one statistically significant motif (**Fig 2A**). The competitors with high affinity contain one motif, 5’-GTGGCAGT-3, while the competitors with low affinity have a similar but different motif, 5’-TGGCACnC-3. Comparison indicated that the identified two motifs share a small motif, 5’-TGGC-3. This makes sense as these two sets of competitors were initially identified based on the inclusion of this motif within their sequences. However, comparison revealed two major differences between the two motifs. First, the competitors with high affinity contain G as a preferred base at the 1^st^ position whereas the competitors with low affinity do not include this preferred base at their 1^st^ position. Instead, their motif starts with a T base, which corresponds to the 2^nd^ position of the motif derived from the competitors with high affinity. Second, the competitors with high affinity have a T base at the 8^th^ position whereas the competitors with low affinity do not have any preferred base at that particular position. We predicted that these two differences are likely responsible for the two different binding affinities exhibited by the two sets of competitors. To test this possibility, we performed another series of gel shift assays using two individual competitors with high affinity (**Fig 2B**). Both competitors contain TGGC and T in the respective positions, which are underlined. However, the first oligonucleotide duplex from *Slc38a2* has T at the 1^st^ position instead of G, whereas the second duplex from *Slc38a4* has G at the 1^st^ position. As predicted, the first duplex did not compete as well as the second duplex against the Pgm2l1 probe, showing much higher levels of PEG3 binding to the Pgm2l1 probe. We also mutated the two positions within these oligonucleotide duplexes: the first position, GG within TGGC, was mutated to AA while the T at the 8^th^ position to C. The mutated duplexes were tested against the Pgm2l1 probe along with the original duplex. As shown in **Fig 2B**, the mutations on both positions were very effective in reducing the binding affinity of PEG3 although the GG-to-AA mutation in the TGGC motif appeared to have more dramatic effects on the binding than the T-to-C mutation in the 8^th^ position. Overall, this series of analyses identified a DNA-binding motif, 5’-GTGGCAGT-3’, showing high levels of binding affinity to the protein PEG3. The results also demonstrated the significant roles played by the four positions in PEG3 binding: the G base at the 1^st^, 3^rd^, 4^th^ positions and the T base at the 8^th^ position.

### Comparison of the new and previous DNA-binding motifs of PEG3

**Fig 3:**
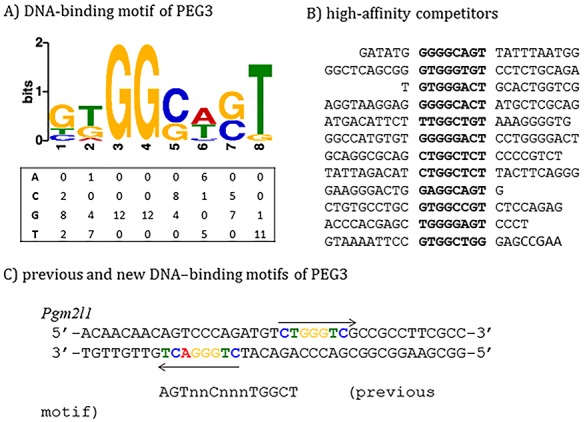
Consensus DNA-binding motif of PEG3. (**A**) The newly derived DNA-binding motif is presented with a tabulated matrix. This matrix has been derived from the sequence alignment of 12 competitors with high affinity shown in (**B**). The new motif was also compared with the previously defined motif of PEG3 using the sequence of the Pgm2l1 oligonucleotide duplex (**C**). Two separate motifs localized in an opposite direction are indicated with two arrows.

The newly identified DNA-binding motif of PEG3 was further analyzed as described below. First, we have derived a matrix using the sequences of 12 competitors with high affinity (**Fig 3A and 3B**). According to the tabulated matrix, the following features are critical for PEG3 binding. Three positions within this motif are absolutely critical: G (3^rd^), G (4^th^), and T (8^th^). The G base is preferred at the 1^st^ position. The two positions, 5^th^ and 7^th^, are also preferred by either G or C, but not by A or T. It is, however, important to note that the relative contribution of a preferred base at each position to the overall binding affinity of PEG3 is currently unknown. As such, several tested duplexes do not contain the preferred bases at all of the critical positions, yet they still display high levels of the DNA-binding affinity, as seen in the case of Paqr7 and Pik3c3 (**Fig 1A**). Thus, these features could be a set of good, but not necessarily perfect, indicators for predicting the potential target sites of PEG3. Second, this new motif was further compared with all the known DNA-binding motifs deposited in the JASPAR database using the TOMTOM program [20]. According to the results, this new motif does not overlap with any known motifs although it shows some levels of similarity to the following known motifs: CRZ1 (GnGGCTnnG) and MYB (TGGCAGTTG). Also, genome-wide scan revealed that around 84,000 loci of the mouse genome contain this motif, and that some of these loci are associated with the genes involved in the following biological pathways, calcium ion binding, organ morphogenesis, proteinaceous extraceullar matrix, glucose homeostasis, and extracellular space. Finally, this new motif was compared with the previously reported motif of PEG3 (**Fig 3C**). Careful inspection revealed that the previously reported motif, 5’-AGTnnCnnnTGGCT-3’, from *Pgm2l1* covers the region that may have two separate motifs, each of which shows some levels of similarity to the new motif. These two motifs are localized 4 bp apart in an opposite direction: the first one, 5’-CTGGGACT-3’, and the second one, 5’-CTGGGTCG-3’. The first one has all the preferred bases at the critical positions: GG at the 3^rd^ and 4^th^ positions and T at the 8^th^ position. In contrast, the second one lacks the T base at the 8^th^ position. The Pgm2l1 nucleotide duplex has been a very consistent probe in the past, displaying high levels of DNA-binding affinity to the protein PEG3 [10]. We surmise that the presence of two separate motifs within the small region of the Pgm2l1 probe may be responsible for the observed high levels of the binding affinity. Independent surveys also revealed that other target regions seem to have a similar feature, the presence of two separate motifs within their genomic regions. Although premature, this might be another feature for high-affinity binding sites of PEG3. Taken together, the above results demonstrated several key features that are associated with a new DNA-binding motif for PEG3.

### *Slc38a2* and *Slc38a4* as potential downstream genes of *Peg3*

**Fig 4:**
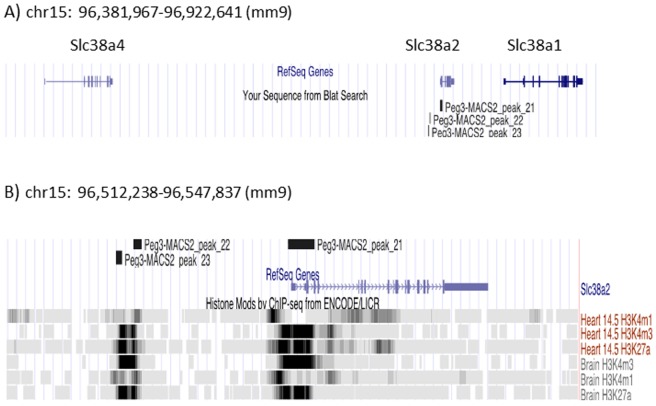
Genomic locus containing *Slc38a2* and *Slc38a4*. (**A**) The 500-kb genomic region containing solute carrier family 38 in mouse chromosome 15 is presented with the image derived from the UCSC genome browser. Small vertical lines indicate the exons of each gene. (**B**) The magnified view of *Slc38a2* is presented with histone modification profiles. The promoter and 5’-side enhancer of *Slc38a2* are modified with high levels of H3K4me1, H3K4me3 and H3K27ac. These two regions have also been identified through PEG3 ChIP-Seq as indicated by the positions of three ChIP-Seq peaks.

According to the results (**Table 1**), PEG3 may be involved in transcriptional control of one particular gene, *Slc38a2*, since three ChIP-Seq peaks are all associated with this gene (**Fig 4B**). This gene is localized in the middle of a gene cluster that spans the 500-kb genomic distance in mouse chromosome 15 (**Fig 4A**). This cluster contains three members of the solute carrier family 38: *Slc38a1*, *Slc38a2* and *Slc38a4*. Among these three genes, two genes are noteworthy for our results, *Slc38a2* and *Slc38a4*. *Slc38a2* is expressed ubiquitously in various tissues whereas *Slc38a4* is highly expressed in placenta and during embryogenesis [21]. Also, *Slc38a4* is imprinted with its paternal allele-specific expression in the majority of fetal and adult tissues except liver [22]. It has been hypothesized that some imprinted genes might control other imprinted genes as part of imprinted gene networks [23]. Therefore, we decided to follow up potential binding of PEG3 to both genes, *Slc38a2* and *Slc38a4*.

**Fig 5:**
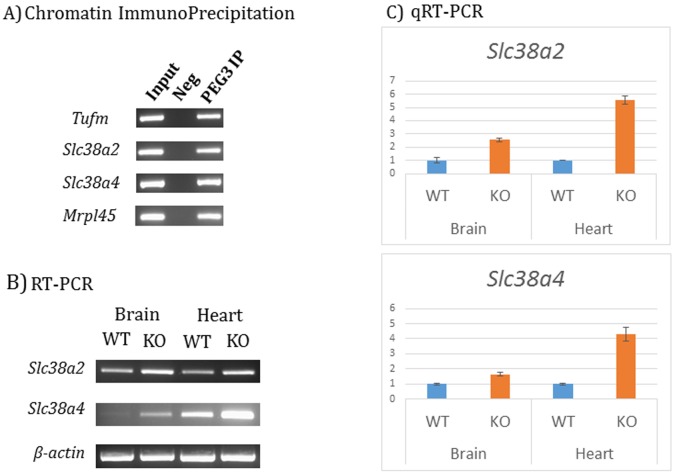
ChIP and qRT-PCR analyses on *Slc38a2* and *Slc38a4*. (**A**) Individual ChIP experiments were performed to further confirm *in vivo* binding of PEG3 to several candidate regions using the chromatin prepared from neonatal brains. The immunoprecipitated DNA was used as templates for PCR amplification. The results of these ChIP experiments were presented in the following order: Input, Neg and PEG3 IP. Each tested locus was indicated with its gene name on left. (**B**) RT-PCR analyses using the total RNA isolated from the brains and hearts of wild-type (WT) and knockout (KO) littermates for *Peg3*. The observed changes in the expression levels of *Slc38a2* and *Slc38a4* were further analyzed using qRT-PCR (**C**). For this series of analyses, we used the *β-actin* as an internal control.

As an initial step, we performed individual ChIP experiments using the chromatin prepared from neonatal brains (**Fig 5A**). The prepared chromatin was immunoprecipitated with polyclonal antibody against PEG3. The promoter regions of both genes were tested using the immunoprecipitated DNA. As shown in **Fig 5A**, high levels of the enrichment were observed from both promoters. We also tested other targets detected through ChIP-Seq experiments, including *Tufm* and *Mrpl45*, which also showed similar levels of the enrichment. These results confirmed the *in vivo* binding of PEG3 to these regions. We also tested potential roles of PEG3 in the transcriptional control of the two genes, *Slc38a2* and *Slc38a4*. For this purpose, we used one mutant allele of *Peg3*, termed CoKO allele [5,24]. This mutant allele with paternal transmission is known to truncate the transcription of *Peg3*, abrogating the majority of the protein PEG3. We first isolated total RNA from the brain and heart of two one-month-old littermates: a wild type (WT) and heterozygote for the mutant allele (KO) (**Fig 5B**). Later, we performed a series of qRT-PCR to test the mutational effects of *Peg3* on the transcriptional levels of the two genes. It is important to note that the expression levels of *Slc38a2* are relatively high in both brain and heart as compared to those of *Slc38a4*, which is consistent with the fact that *Slc38a2* is expressed more ubiquitously in various tissues than *Slc38a4* [21]. Nevertheless, comparison of the expression levels between WT and KO indicated an overall up-regulation of both *Slc38a2* and *Slc38a4*: 2.5 and 5.5 fold up-regulation of *Slc38a2* and 1.5 and 4.0 fold up-regulation of *Slc38a4* in brain and heart, respectively (**Fig 5C**). This observed up-regulation suggests that PEG3 may function as a transcriptional repressor for both genes. This is consistent with the previous observation from other target loci, including *Pgm2l1* and placenta-specific gene families [5,10].

**Fig 6:**
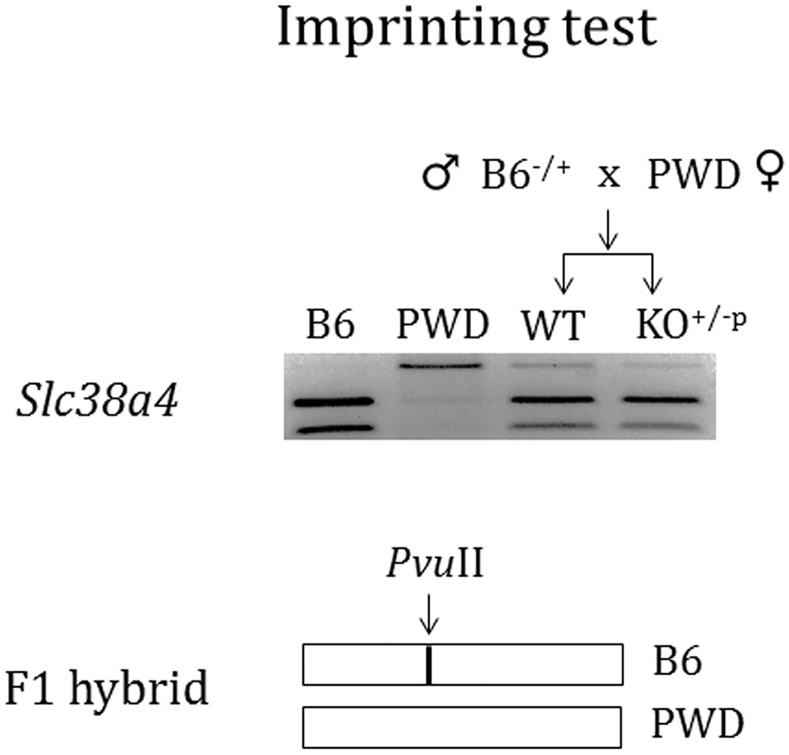
Imprinting test of *Slc38a4*. The paternal allele-specific expression of *Slc38a4* was tested using the F1 hybrid derived from the interspecific crossing of a male C57BL/6J (B6) heterozygous for Peg3 KO and a female PWD/PhJ breeder. The differentiation of two parental alleles was visualized through a restriction enzyme (*Pvu*II) digestion on the RT-PCR products that had been derived from the transcribed region of *Slc38a4*. The schematic representation is shown on the bottom panel. The paternal allele-specific expression was detected in both WT and KO, indicating that the observed up-regulation of *Slc38a4* in Peg3 KO involves only the active paternal allele, but not the imprinted maternal allele.

Given the fact that *Slc38a4* is imprinted, we further characterized the observed up-regulation in terms of its imprinting status (**Fig 6**). This series of analyses were performed using the total RNA isolated from an F1 hybrid, which was prepared through the interspecific crossing of a male Peg3^CoKO^ heterozygote with the C57BL6/J background and a female breeder with the PWD/PhJ background. A SNP (Single Nucleotide Polymorphism) between the two subspecies was identified within the transcribed region of *Slc38a4*, and this SNP was subsequently visualized through a restriction enzyme digestion, as shown in the schematic representation in **Fig 6**. This series of imprinting tests confirmed that there was no change in the imprinting status of *Slc38a4*. This gene is still expressed mainly from the paternal allele regardless the presence (WT) or absence (KO) of PEG3. This suggests that the observed up-regulation of this gene in the tissue samples of the Peg3-KO mutant may involve only the paternal active allele without any contribution from the maternal imprinted allele. Taken together, these results demonstrated that PEG3 directly binds to both *Slc38a2* and *Slc38a4*, and suggest that PEG3 might function as a transcriptional repressor for both genes.

## Discussion

In the current study, we reanalyzed the previous ChIP-Seq results and also redefined the DNA-binding motif of PEG3. According to the results, PEG3 may bind to a subset of genes that are closely associated with the known functions of *Peg3*. A newly defined DNA-binding motif of PEG3, 5’-GTGGCAGT-3’, displays several features that are critical for the binding of PEG3. This motif also provides a tabulated matrix that can be used for predicting other unknown genomic targets. We further analyzed the two loci, *Slc38a2* and *Slc38a4*, among the newly identified set of genomic targets. The results indicated that PEG3 likely functions as a transcriptional repressor for these two loci.

The results and subsequent interpretation described in the current study are different from those described in the previous study although a same set of ChIP-Seq results was analyzed in both cases. One of the main culprits for these differences stems from our earlier handling of ChIP-Seq results, which was incomplete due to the lack of proper controls. Subsequently, including additional ChIP-Seq sets from the preimmune serum and input DNA resulted in a dramatic improvement in predicting potential binding sites (or peaks), removing the vast majority of inaccurate peaks. As an outcome, a handful of genes have been identified as potential targets of PEG3 (**S1 Table** and **Table 1**). Despite this small number of targets, we believe that this set of targets are most likely genuine *in vivo* targets of PEG3. This prediction is further supported by the following observations. The 21 targets are all localized within either the promoters or enhancer regions of the associated genes. Furthermore, the functions of these genes are very closely connected to the known functions of *Peg3*. For instance, *Peg3* is involved in energy expenditure [5,25], yet several targets of PEG3 are localized in the promoters of genes that play important roles in mitochondrial function. This set of genes includes *Tufm* (elongation factor Tu, mitochondrial isoform 1), *Mrpl45* (39S ribosomal protein L45, mitochondrial) and *Slc25a29* (mitochondrial carnitine/acylcarnitine carrier). The second set of examples includes the two genes that are involved in controlling circadian rhythm, *Cry2* (cryptochrome-2) and *Per1* (period circadian protein homolog 1). The proteins encoded by these two genes are known to form a complex controlling transcriptional control for a large number of genes in the brain [17,26,27]. Interestingly, both genes are predicted to be downstream genes of *Peg3* based on the observation that PEG3 binds to the promoters of these genes. Overall, we believe that the results and new interpretation described in the current study most likely reflect the *in vivo* binding and function of PEG3.

The newly defined DNA-binding motif of PEG3 is slightly different from the previous motif (**Fig 3**). This new motif is 8 bp in length, which is shorter than the 14-bp-long previous motif. Despite its shorter length, the new motif is better defined than the previous degenerate one. Within this 8-bp-long motif, each position has a preferred base, and also the degree of this preference has been tabulated as a matrix (**Fig 3A**). Thus, the probability of a given site’s binding to PEG3 can be calculated with this matrix, which eventually helps future studies by other investigators. In terms of PEG3 binding, it is important to note that several targets tend to have two separate motifs within small regions, as seen in the Pgm2l1 probe (**Fig 3C**). It is well known that DNA-binding sites tend to be clustered in small regions [28,29]. Therefore, this might also be the case for PEG3 where two or three binding sites of PEG3 are clustered together. Thus, it would be interesting to test this possibility with a set of newly identified target loci of PEG3 in the near future.

Among the newly identified targets, two genes, *Slc38a2* and *Slc38a4*, have been further analyzed in the current study (**Figs 4–6**). Individual ChIP experiments indeed confirmed the *in vivo* binding of PEG3 to the promoters of these two genes. Also, expression analyses further demonstrated that these genes are up-regulated in the tissues of the KO animals lacking PEG3, suggesting that PEG3 may act as a transcriptional repressor for both genes. Functional connection of *Peg3* to these genes is intriguing given the following reasons. First, it is well known that many imprinted genes are involved in controlling fetal growth rates. These two genes are known to be very important in controlling neutral amino acid transport in major organs, especially in placenta [21,30]. Thus, it makes sense that *Peg3* controls the other genes, such as *Slc38a2* and *Slc38a4*, which are critical for nutrition transport. Second, it has been hypothesized that imprinted genes are connected to each other as part of so called ‘imprinted gene network’ [23]. *Slc38a4* is an imprinted gene that is highly expressed in placenta and also during early embryogenesis. The current study provides a set of results suggesting that this gene is likely controlled by *Peg3*, another imprinted gene. A similar case has also been reported where *Peg3* controls another imprinted gene *Zim1* [11]. Thus, these observations appear to be in agreement with the imprinted gene network model. In that regard, it is interesting to note that only a few imprinted genes can function as DNA-binding proteins, such as *Peg3* and *Zac1*. Furthermore, the proposed imprinted gene network model places *Peg3* at the center of this hub-like network, suggesting that *Peg3* may play a very significant role in the regulation of multiple imprinted downstream genes. Consistent with this prediction, the previous genome-wide expression analyses also indicated that the KO placenta lacking PEG3 display changes in the expression levels of several imprinted genes, including *Dlk1*, *Phlda2* and *Ascl2* [5]. Thus, it will be very interesting to investigate this aspect of *Peg3* in the near future.

## Materials and Methods

### Ethics Statement

All the mouse experiments were performed in accordance with National Institutes of Health guidelines for care and use of animals and also approved by the Louisiana State University Institutional Animal Care and Use Committee (IACUC), protocol #13–061. The mouse used for the current study is a mutant strain with C57BL/6N background, and has been maintained in the Lab since 2011 [5]. The current study used about 20 mice, which were humanely euthanized by placing them in an atmosphere of 100% CO_2_ for 10 minutes, followed by bilateral open thoracotomy.

### ChIP-Seq Analysis

ChIP-Seq experiments were performed using the brain extract prepared from a 3-month-old male mouse according to a previously described protocol [10]. A portion (one-half) of the crosslinked and sonicated brain extract was immunoprecipitated with the PEG3 antibody, the quality of which has been tested and confirmed through previous studies [10,31]. For the sequencing analysis, a DNA library of the immunoprecipitated DNA was produced using the Ion Xpress Fragment Library Kit (Life Technologies). Following Life Technologies’ instructions, ChIP DNA was enzymatically sheared to approximately 100–200 bp in length, size-selected with AMPure XP beads (Beckman Coulter), ligated with the supplied DNA adapters, and amplified by PCR for 10 cycles. The ChIP-Seq library was sequenced using the Ion PGM Sequencer (Ion Torrent technology). Raw sequence reads from the runs (~3.3 million) were aligned to the mouse reference genome (mm9) using Bowtie2 [32]. This series of ChIP-Seq experiments were also repeated using the input DNA and the immunoprecipitated DNA with a preimmune serum, generating 4.4 and 4.2 million reads, respectively. These two sets of reads were also mapped using Bowtie2. The three sets of sam files were converted into the corresponding bed files using samtools. Finally, the bed files were used for predicting peaks using the callpeak function of MACS2 [33]. We individually tested the input and preimmune sets as a control for predicting peaks with the cutoff *p* value at 1.00e-05. There were no major differences between these two independent peak callings except the fact that about 10% of the peaks (8 out of 81 peaks) were detected only in one library due to artificial pileup. These artificial peaks were indicated in **S1 Table**. Thus, the current study used the set of peaks that had been predicted using the preimmune set as a control.

### Gel shift assay

Gel shift assay was performed using the Gel Shift Assay System (Promega, Cat. No. E3050). Briefly, 5x binding buffer, 2.72 μg mouse brain nuclear extract (Active Motif, Cat. No. 36053), 0.7 pmol unlabeled competitor duplex were first incubated at room temperature for 10 mins. Afterwards, 0.007 pmol [γ-32P]-labeled Pgm2l1 duplex probe was added and incubated for additional 20 mins. The entire reaction mixture was loaded on a 5% TBE gel (Bio-Rad, Cat. No. #456–5014), and later exposed to a film for 12 hrs at -80°C before developing.

### Chromatin Immunoprecipitation

Individual ChIP experiments were performed using neonatal mouse brains. The homogenized samples were fixed in 1% formaldehyde for 20 mins, and then resuspended in the lysis buffer containing the protease inhibitor cocktail (Millipore, Cat. No. 539131). The released nuclei were sheared with sonication to generate the DNA fragments with sizes ranging from 300 to 500 bp in length. Immunoprecipations were performed with the custom-made antibody and also with the commercial polyclonal anti-PEG3 antibody (Abcam, Cat. No. ab99252). The complexes were pulled down using Protein A/G PLUS-Agarose beads (Santa Cruz, Cat. No. sd-2003). Immunoprecipitated DNA was de-crosslinked, purified with phenol/chloroform, precipitated, and finally dissolved in 50 μl 1xTE for PCR.

### RNA isolation and qRT-PCR Analysis

Total RNA was isolated from the brains and hearts of WT and Peg3-KO mice using Trizol (Ambion, Cat. No. 15596018). The subsequent RNA was used for cDNA synthesis with M-MLV reverse transcriptase (NEB, Cat. No. M0253L). Quantitative RT-PCR was performed using the SYBR Select Master Mix (Applied Biosystems, Cat. No. 4472908), and analyzed on the ViiA 7 Real-time PCR system (Life Technologies). All qRT-PCR were performed using standard PCR conditions with β-actin as an internal control [10,31]. The information regarding oligonucleotides used for qRT-PCR is available (**S2 File**).

## Supporting Information

S1 File The list of competitors used for gel shift assays. These sequences have been initially identified from the genomic targets of PEG3 based on the presence of TGGC motif within their sequences.
(DOCX)

S2 File The list of oligonucleotides used for ChIP, qRT-PCR and imprinting tests. (DOCX)

S1 Table The list of genomic targets predicted through ChIP-Seq experiments. This list has been derived from the output file of the callpeak function of MACS2. The peaks marked in red are thought to be generated due to artificial pileup during bioinformatics processing of ChIP-Seq experiments, thus need to be regarded as artifacts.
(XLSX)
