# Supplementary material for: DNA-Binding Motif of the Imprinted Transcription Factor PEG3
Source: PLoS One. 2015 Dec 21;10(12):e0145531. doi: 10.1371/journal.pone.0145531 (PMC4686966; doi:10.1371/journal.pone.0145531)
Supplement: S1 File — These sequences have been initially identified from the genomic targets of PEG3 based on the presence of TGGC motif within their sequences. (DOCX) [file pone.0145531.s001.docx]

**Competitors with high affinity**

>Slc38a2-2_EMSA_F

CACCCCTTTACAGCCAAAGAATGTCATGGTCGGTGT

>Slc38a2-4_EMSA_F

AGGGACTCCCCAGCTCGTGGGTTGTCTTGCTAAATCC

>Slc38a4-2_EMSA_F

CTCCTATTCGAACTGTGCCTGCGTGGCCGTCTCCAGAG

>Ric8b_EMSA_F

GATGGCCCTGAAGTAAGAGCCAGATGTCTAATAAAGTTT

>Pik3c3_EMSA_F

TTGATGAATTCAGTAAAATTCCGTGGCTGGGAGCCGAA

>Paqr7_EMSA_F

CTGCCTGGCTCAGCGGGTGGGTGTCCTCTGCAGACT

>Tufm_EMSA_F

GATATGGGGGCAGTTATTTAATGGCCAAGGACTGGA

>Mrp145-1_EMSA_F

GCCTCCTGCGCAGGCGCAGCTGGCTCTCCCCGTCT

>Malat1-1_EMSA_F

AGTCCCCAGGAGTCCCCCACACATGGCCCCAGGCTGGT

>H19-EMSA-1_F

AGTGGCTGGTAAGACCGAAGTTGCCGAGCAGCGACCA

>Oxtr_BS1_F

CACTGCCTCCAGTCCCTTCTCTCCCTGAAATCATCTCT

>Oxtr_BS3_F

TCCCGCCTGCGAGCATAGTGCCCCCTCCTTACCTCCCACCCA

**Competitors with low affinity**

>Slc38a2-1_EMSA_F

CGGGGCACTGCAGCCCCGTGGGTGGCCGCCACCCGCG

>Slc38a2-3_EMSA_F

AGGGACTCCCCAGCTCGTGGGTTGTCTTGCTAAATCC

>Slc38a4-1_EMSA_F

CAGGTTCCTGCACTGCTATTGGTGGCTAGGCTGGAAG

>Plk5_EMSA_F

AGTTTAGTGCCAGCCATGGGAATGGCTCTGTTCCACT

>Slc25a29_EMSA_F

AGACGGTGCCAAGGATGTGCCTGTGTCTTTAAAGCAAC

>Saysd1_EMSA_F

CTCGAGAGGAGAGTGAACTGAGTGTCCCATCTCCTCC

>Mix1_EMSA_F

TAATAGCTCAAAGTTGCTGTGTGTGTCTAGTGGCTCTC

>Per1_EMSA_F

CTGATTTAGGCAGGGCGGGGTTGTCTCTGCAGCCAG

>Mrp145-2_EMSA_F

GATGTGAATACAGGTTATTAATTGGCTTGTGGCAAAA

>Skp2-1_EMSA_F

GAAGTGGGTGGAGCTCCGGCTGGCTGGGAAATGTC

>Skp2-2_EMSA_F

GGGGGAGTTCCAGATGGTGAGTGGCCGCCGCCGCCA

>Malat1-2_EMSA_F

TGATCTGCTTAGCACAGACCCTGGCTCGAGGGATGG
