# Supplementary material for: DNA-Binding Motif of the Imprinted Transcription Factor PEG3
Source: PLoS One. 2015 Dec 21;10(12):e0145531. doi: 10.1371/journal.pone.0145531 (PMC4686966; doi:10.1371/journal.pone.0145531)
Supplement: S2 File — (DOCX) [file pone.0145531.s002.docx]

**PCR primers for ChIP experiments**

Tufm_ChIP F : CAAGCGTCCAAAGGACTATTG

Tufm_ChIP R : GATCTCCCAGCCTGTAAGAG

Mrp145_ChIP F : CTTCTGATTTCAGAGGGAAGC

Mrp145_ChIP R : GTGCGCAATGAAACCACTTTG

Slc38a2_ChIP F : TCGTGATCATCAGGGGTAATC

Slc38a2_ChIP R : GAAGCACCGCGCAAAGCTTC

Slc38a4_ChIP F : GTGACTAGAAGTGAAGTAAACCAG

Slc38a4_ChIP R : CAGCTGCTATCGTTCAAGGAG

**PCR primers for qRT-PCR experiments**

Slc38a2 RT F : CCTCAAGACTGCCAACGAAG

Slc38a2 RT R : GAAGAGGTAGCTTGACATAGCC

Slc38a4 RT F : CAGTGGGATCTTAGGCTTGTC

Slc38a4 RT R : GCCATCCAAATGCTTTCTCG

**PCR primers for imprinting tests**

Slc38a4 imprinting F : TAAGATCACCCCAGAAGATTG

Slc38a4 imprinting R : CCATGATAGAGCTAGAAAAACG
